# Neural entrainment to the beat and working memory predict sensorimotor synchronization skills

**DOI:** 10.1038/s41598-025-93948-9

**Published:** 2025-03-26

**Authors:** María de Lourdes Noboa, Csaba Kertész, Ferenc Honbolygó

**Affiliations:** 1 Doctoral School of Psychology, ELTE Eötvös Loránd University, Budapest, Hungary; 2 Institute of Psychology of the Faculty of Education and Psychology, ELTE Eötvös Loránd University, Izabella u. 46, Budapest, 1064 Hungary; 3 Brain Imaging Centre, Research Centre for Natural Sciences, Budapest, Hungary

**Keywords:** Neural entrainment, Rhythm, Sensorimotor synchronization, EEG, Frequency tagging, Human behaviour, Cognitive neuroscience

## Abstract

Neural entrainment to rhythmic patterns has been proposed as a mechanism that underlies beat perception and could explain individual differences in sensorimotor synchronization abilities. Nevertheless, the neural and cognitive mechanisms behind beat perception remain an active research area. Our study examined whether neural entrainment to rhythmic patterns, cognitive resources, specifically working memory and musical background predict sensorimotor synchronization skills in adults. Using electroencephalogram (EEG), we recorded steady-state evoked potentials (SS-EPs) while participants passively listened to short tone sequences featuring syncopated (tones missing from certain beats) and unsyncopated (tones present on every beat) rhythms. Participants also completed a finger-tapping task, measuring tapping consistency and asynchrony, and a counting span task to assess working memory. Results showed increased steady-state evoked potentials (SS-EPs) at beat-related frequencies (1.25 Hz and its harmonics, 2.10/2.50 Hz, 5 Hz), indicating faithful neural tracking of the rhythms. Contrary to expectations, stronger neural entrainment to unsyncopated rhythms was associated with greater tapping variability and lower synchronization accuracy. In contrast, working memory capacity positively predicted tapping consistency, suggesting that automatic beat-based predictions as reflected in neural entrainment may reduce the flexibility needed for rhythm production. Musical background was not a significant predictor of tapping performance, while working memory was suggesting that working memory capacity support rhythm production skills by maintaining internal representations of time intervals. Our results challenged the assumption that stronger neural entrainment universally enhances synchronization skills and highlighted the multidimensionality of rhythm processing, and the complex relationship between neural entrainment, cognitive resources, and sensorimotor synchronization skills.

**Supplementary Information:**

The online version contains supplementary material available at 10.1038/s41598-025-93948-9.

## Introduction

Music has been part of the history of humanity since its beginning^1^; synchronizing one’s movement to music through clapping and dancing feels so natural that we overlook how highly complex it is^2–4^. Sensorimotor synchronization, the ability to align motor actions with auditory information relies on the perception of the “beat”^5^, we group events based on their salience as they unfold over time, building metrical hierarchies with patterns of stronger and weaker beats that would allow us to make predictions of the following temporal events or “beats” to come to^6–8^. Therefore, understanding the neural and cognitive mechanisms that support beat perception and synchronization remains an area of active research^9^.

In the present study, we aim to identify the predictors of sensorimotor synchronization skills in adults. Specifically, we focus on neural entrainment to rhythmic patterns with unsyncopated and syncopated beats, as well as the role of cognitive resources—particularly working memory—and musical background.

Beat perception differs from implicit learning skills, such as sequential learning, as it occurs even in the absence of attention and when controlling for transitional probabilities and pattern learning^10^. Beat perception has also been reported in infants^11,12^ and newborns^13^. Although studies have shown that beat perception is not a uniquely human ability^14,15^, it is essential for music production and perception^6,16^.

One important neural mechanism proposed to aid beat perception is neural entrainment, which is defined as the temporal alignment of endogenous neural oscillatory activity and an exogenous rhythmic stimulus^17^. Neural oscillations in the delta (1–3 Hz) and beta (15–30 Hz) bands have been linked with beat perception^9,18–20^. Behavioral synchronization tasks have shown that participants have better tapping synchronization at beat tempo ranges of 500–600 ms (approximately 2 Hz), a seemingly preferred tempo range that coincides with delta band rhythms^5,14,21^. Exploiting this preferred rhythm, neural responses in the low-frequency band faithfully track the rhythmic contour of the external stimulus, matching the phase of neural oscillations to the more salient event and predicting essential events to come. The beat, or in this sense, greater sustained cortical tracking to the beat, is associated with more significant neural responses to the beat and meter-related frequencies; evidence for this has been found in studies using the frequency tagging approach^12,22,23^. Frequency tagging is an EEG method that quantifies Steady-state Evoked Potentials (SS-EPs) to obtain the amplitude of frequencies of interest based on the rhythm envelopes^24–27^. Previous SS-EPs studies have found enhanced neural responses at beat frequency (1.25 Hz) and related meter frequencies (0.43, 2.50, and 5 Hz) compared with nonrelated beat and meter frequencies. This neural response is independent of tempo^27^, and it has been found in infants and adults alike^12^.

More importantly, this enhanced cortical response at 1.25 Hz has been considered the result of internal beat generation. Nozaradan and colleagues^23^, distinguishing between syncopated rhythms, in which the tones do not coincide with the beat, and unsyncopated rhythms, where the tones coincides with the beat, showed that both tone sequences elicit SS-EP amplitudes at beat frequency and related meters in both rhythms in comparison with nonrelated beat frequencies. The authors suggest that there is selective neuronal entrainment to the beat and meter present even in sound patterns where there is no predominant acoustic energy at the beat frequency, such as in the case of syncopated rhythms.

Bower and collaborators^28^ have differentiated between two types of predictions for processing rhythmic stimuli. Beat-based expectations are usually given by a regular isochronous rhythm, allowing the detection of a “beat” or “pulse.” In contrast, memory-based or pattern-based expectations come from learning associations between a cue, a temporal interval, and the occurrence of an event or target, as well as learning sequences of temporal structures^29^.

We encounter various types of temporal regularities in the environment, which may be processed and exploited by different neural mechanisms in diverse ways. Beat-based regularities, which arise from isochronous, highly periodic rhythms that are explicitly guided by the beat, elicit the entrainment of neural oscillations, resulting in enhanced neural responses that are phase-locked with the expected beat, as suggested by neural entrainment models and the dynamic attending theory^23,30,31^.

While memory-based predictions are guided by the association of cues and temporal intervals that build a stable pattern, attentional and memory resources support pattern building, but once built, fewer attentional and memory resources are needed; therefore, there is attenuation of sensory responses for expected events. This is in line with predictive coding theories: increased sensory responses would be present for unexpected events, as attention and top-down mechanisms would need to readjust and adapt to a new pattern, exerting more resources to accomplish it^32,33^.

Beat-based expectations are guided by stimuli with an embedded temporal structure, whereas memory-based expectations are based on prelearned associations^34^. The first reflects exogenous temporal expectations, whereas the latter reflects endogenous expectations. This distinction also requires reflection on the dissociation between automatic and intentional processes. Exogenous temporal expectations drive automatic processes supported by the entrainment of neural oscillations. In contrast, endogenous temporal expectations involve intentionality and are associated with attenuation of the alpha band (8–12 Hz) in anticipation of expected events^35^.

Bouwer and collaborators^36^, in a study including silent periods after the presentation of nonisochronous rhythms have shown that beat-based predictions lead to persistent neural entrainment after stimulus cessation. and this enhancement was not present during silence for memory-based sequences or random sequences. This suggests that neural entrainment supported by beat-based predictions was sustained and outlasted rhythmic stimulation, providing further evidence that the neural entrainment of low-frequency oscillations supports beat perception.

Neural entrainment to the beat, alongside fine-auditory processing and sensorimotor synchronization have been suggested to be common mechanisms for rhythm processing^8^; therefore, we could assume that there is a positive association between neural entrainment to the beat patterns and sensorimotor synchronization skills. Previous studies have shown that the stronger the sustained entrainment is, the better synchronization participants have in behavioral sensorimotor synchronization tasks, for example, in finger tapping to the metronome or music^21,27,37^. A study by Nozaradan and colleagues^27^, indeed showed that participants with stronger neural entrainment at the beat frequency also had improved synchronization accuracy in a finger-tapping task as well as better temporal prediction abilities, allowing them to adequately anticipate important events in the tone sequences, guiding their motor planning to synchronize their movements with complex temporal structures. This suggest that individual differences on beat-based predictions evidenced on neural entrainment to the beat patterns can explain individual differences in rhythmic processing skills^28^.

Previous studies have also revealed an association between musical background and an enhanced performance on sensorimotor synchronization tasks^38,39^, as well as enhanced neural entrainment in musicians compared with nonmusicians^40^; however, more studies are needed to identify how these musical background alongside neural entrainment patterns impact behavioral performance in a musical context.

Another important caveat is the role of other cognitive factors, such as working memory as defined by Baddeley and Hitch^41^, in supporting rhythm processing skills, as previous studies have found a positive correlation between working memory and rhythm production performance^42–44^. A study with school-age children identified working memory and tapping consistency as predictors of rhythm discrimination abilities: participants with higher tapping consistency and higher auditory counting span were better at discriminating musical rhythms^42^. The suggested connection between working memory capacity and temporal information processing is that temporal intervals have to be encoded and maintained in working memory to be used later as a reference^45–47^, when presented with a tapping task participants have been shown to deploy different strategies to solve the task, for instance, creating a subdivision of a given interval, a strategy that relies on working memory capacity^48^. Therefore, individual differences in cognitive resources such as working memory in addition to musical background and neural entrainment to rhythmic patterns could help us unveiling variability in sensorimotor synchronization skills.

The present study has several aims. First, we aim to replicate the findings that EEG cortical responses faithfully track unsyncopated and syncopated rhythms. Based on the stimuli envelope, we expect to find an enhanced neural response at peaks corresponding to the beat frequency (1.25 Hz) and related harmonics at 0.43, 2.50, and 5 Hz for the unsyncopated rhythm and at 0.43, 2.10 and 5 Hz for the syncopated rhythm. Second, we aim to identify whether neural entrainment alongside cognitive resources, specifically working memory and musical background could predict sensorimotor synchronization skills in adults. We hypothesize that enhanced neural responses, larger SS-EPs amplitudes to both rhythms and high working memory performance will be associated with higher tapping consistency and lower tapping asynchrony. We also hypothesize that participants with a musical background will have a better tapping performance.

##  Materials and methods

### Participants

Thirty-four healthy young adults (Mage = 20.7 years; SDage = 2.9; 5 males, 29 females) participated in this study. All participants were right-handed, had normal or corrected-to-normal vision, and had normal hearing levels according to the audiometry measurements; most of them were undergraduate students. All participants were native Hungarian speakers. Informed consent was obtained from all subjects and/or their legal guardian(s). The experiment was approved by the United Ethical Review Committee for Research in Psychology (EPKEB), Budapest, Hungary, and was conducted following the Declaration of Helsinki.

Two participants were excluded from the analysis because of excessive artifacts (see EEG Recording and Preprocessing), and two participants’ standardized SS-EP amplitude values were above 3 SDs from the mean; therefore, they were labelled as outliers and removed from further analysis. We proceeded with the statistical analysis of the remaining thirty participants.

### EEG task: neural entrainment to music

EEGs were recorded while the participants listened to rhythmic sound sequences featuring syncopated and unsyncopated rhythms. We used a modified paradigm from Nozaradan and colleagues^26^, previously applied to detect steady-state evoked potentials (SS-EPSs) using the frequency tagging method.

**Fig. 1:**
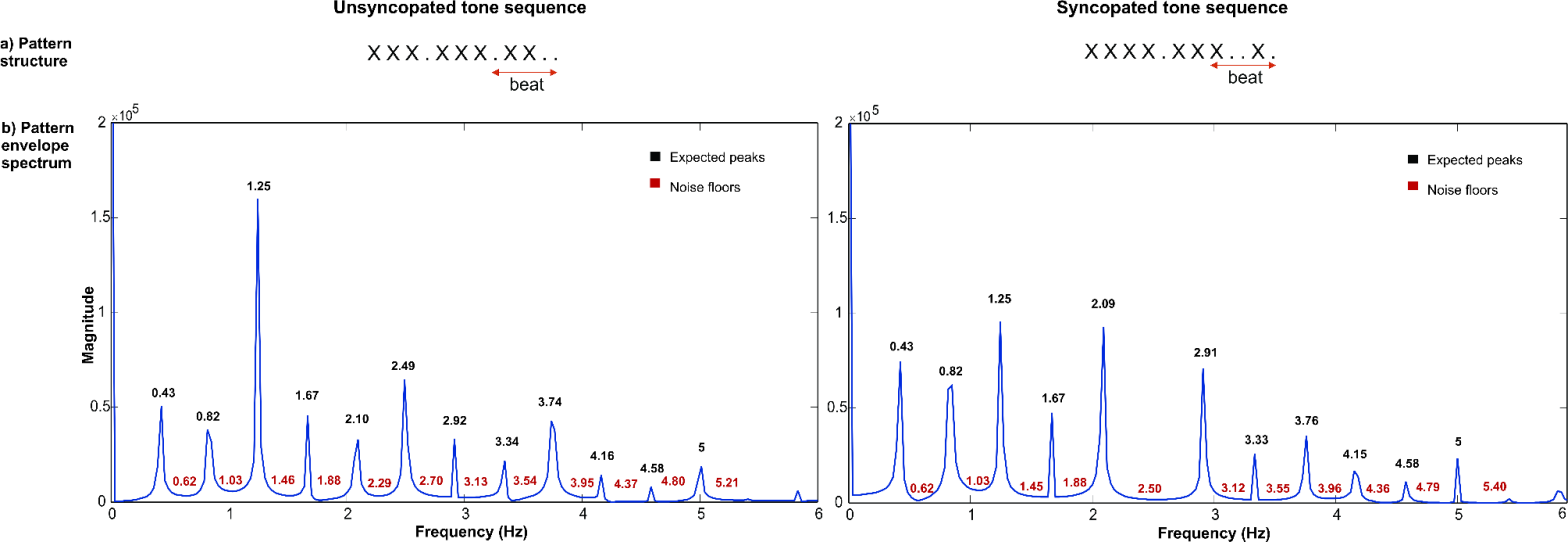
Acoustic signal analysis of unsyncopated and syncopated stimuli. (**A**) Rhythmic patterns, unsyncopated, syncopated. (**B**) Frequencies of interest based on the temporal envelope spectrum of each stimulus. Expected peaks at beat frequency of 1.25 Hz and related harmonics are labelled in black, and noise floors and unexpected peaks are labelled in red.

*Stimuli.* The stimuli consisted of two rhythmic patterns composed of alternating sound and silent intervals (12 events) with a length of 200ms, looping them continuously for 33 s. The sound events consisted of 1000 Hz pure tone sequences, with a rise and a fall time of 10 msec. The sequences were specifically designed to induce the perception of the beat at 1.5 Hz based on a preference for grouping by four events, as represented in Fig. 1. (i.e., either silent or tone events) with three such beats per cycle. The first sequence is a denominated unsyncopated rhythm: the tones coincide with every beat. The second rhythm is syncopated: it includes instances where a tone occurs between beats and is followed by silence on the next beat. The participants were asked to listen to the syncopated and unsyncopated tone sequences presented in a separate block, each rhythmic pattern presented 10 times in each block. Within each block, two of the 10 trials contained a sequence in which the elements were shorter (tempo change). The task of the participants was to decide, at the end of each trial, if the trial contained any kind of change. The order of the presentation of the sequences was counterbalanced and participants were not explicitly told to detect a beat or synchronize to it. The tempo change trials were included to maintain the attention of the participants, but they were not subsequently analysed. Further details of the stimuli can be found in^26,27^.

*Acoustic signal analysis*. A MATLAB script (The MathWorks Inc.) was used to extract the temporal envelope of the two rhythm patterns using the Hilbert function. A fast Fourier transform (FFT) was subsequently applied to compute the frequency spectrum of the acoustic energy. Twelve frequency peaks (0–5 Hz) were identified as frequencies of interest for the unsyncopated stimuli, whereas eleven frequency peaks (0–5 Hz) were identified as frequencies of interest for the syncopated stimuli (Fig. 1).

### Behavioral measures

#### Tapping task

**Fig. 2:**
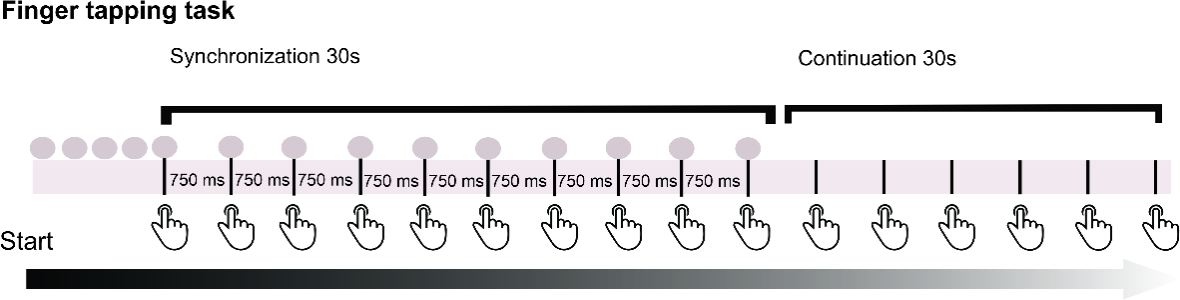
Finger-tapping task procedure. The participants were asked to tap to the beat of a metronome or an instrumental version of a song for 30 s; afterwards, the participants were required to continue tapping for 30 s without stimulus presentation.

We used the finger-tapping task from Kertész and Honbolygó^41^, (see Fig. 2), which was previously used with children. The participants were asked to tap with their dominant hand on an AKAI LPD8 MIDI controller while listening to metronome sounds or music through headphones connected to a computer with a Steinberg U-22 interface. Cubase Elements 12, version 12.0.40 (2022), was used for playback and recording taps.

The metronome condition consisted of stimuli presented at three different tempi (80, 120, 150 bpm), and it consisted of a 30 s woodblock sample from the Cubase5 library. The music condition consisted of three 30 s instrumental versions of three popular songs: Dream, dream, dream (Everly Brothers)—80 bpm, Michelle (The Beatles)—120 bpm, and Johnny B Goode (Chuck Berry)—150 bpm. All three songs were rendered from MIDI scores using virtual instruments with the same instrumentation to avoid timbral differences. The vocal melody and characteristic instrumental parts (e.g. guitar riffs) were removed to avoid the advantages of song familiarity. The participants were given instructions and a short demonstration of the task before the measurements were taken. If a participant misunderstood the task, used an atypical strategy (e.g., tapping in antiphase or double time), or tapped too weakly to be registered by the controller, they were asked to start again after a second demonstration and clarification.

First, to familiarize participants with the MIDI controller, we asked them to spontaneously tap without any stimuli for 30 s approximately; we call this the spontaneous measure trial (SMT). The main task consisted of 6 trials. Three trials were conducted for the metronome and three for the music condition, each presented at three different tempi (80, 120, 150 bpm). Following Kertész and Honbolygó^41^,, participants were required to tap at the same tempo as the stimuli for approximately 30 s until the stimulus had ended. Synchronization accuracy and tapping consistency were calculated in the statistical analysis to measure tapping performance^49^.

#### Counting span task

We used the counting span task^50^ to assess the working memory capacity of participants. Participants were asked to count aloud the blue circles on the computer screen, while yellow circles and blue squares were presented as distractors. After a block (blocks consisting of 2 to 8 different pictures), they were required to recall all the numbers. The task was presented in Presentation software (v. 21.1, Neurobehavioral Systems). A short demonstration was given by the experimenter, and the participant was assured that there was no time limit for counting. The task consisted of three blocks with five sequences of pictures (stimulus sets) in each block. In every block, the first sequence contained two pictures, the second contained three, and so on until the fifth sequence contained six pictures. The participants were required to maintain the final counts of blue circles in each sequence until the end of each block when they were asked to recall them in spoken words in the same order as they appeared. The participant’s counting span for each block was calculated as the highest stimulus set size that was recalled in the correct order^51^. To obtain the final counting span for each participant, we calculated the mean performance across the three blocks. The administration and completion of this task took approximately ten minutes.

#### Other measures

Participants completed a questionnaire that aimed to collect data on their formal musical education. Lacking a standardized questionnaire on the musical background in Hungarian, we asked questions about whether they had received music education, the age of onset, which instrument(s) they play(ed), and the total years of musical education. The mean years of musical education reported was 4.40 years, SD = 4.91 years. The mean number of instruments played was 1.18, SD = 1.21. From our participants, 19 reported to have received musical education, while 11 have not. Lastly, 8 of the participants reported being an active musician, while the rest (*N* = 22) reported to not being active.

To make sure that participants have normal hearing levels, we completed an audiometry measurement. During the measurement, participants were presented simple tones of 250, 500, 1000, 2000, 4000, 6000 and 8000 Hz to both ears through headphones, and they were required to press a button when they detected the sound. Each tone was presented twice. According to the results of the measurements, all participants had an absolute threshold below 20 dB for each frequency.

### Procedure

The experiment session began with participants completing the consent form, Edinburgh handedness questionnaire, and musical background questionnaire; posteriorly, participants went inside a sound-isolated booth, where they took an audiometry test and where the EEG recording would take place. In the first block of the experimental session, EEG data were recorded. The EEG data was recorded during the neural entrainment to the music task. The EEG recording lasted approximately 20 min. Then, the participants had a 15-minute break. In the second block, participants completed behavioral measures, including the tapping task and the counting span task. The entire experimental session lasted approximately 3 h.

### EEG recording and preprocessing

**Fig. 3:**
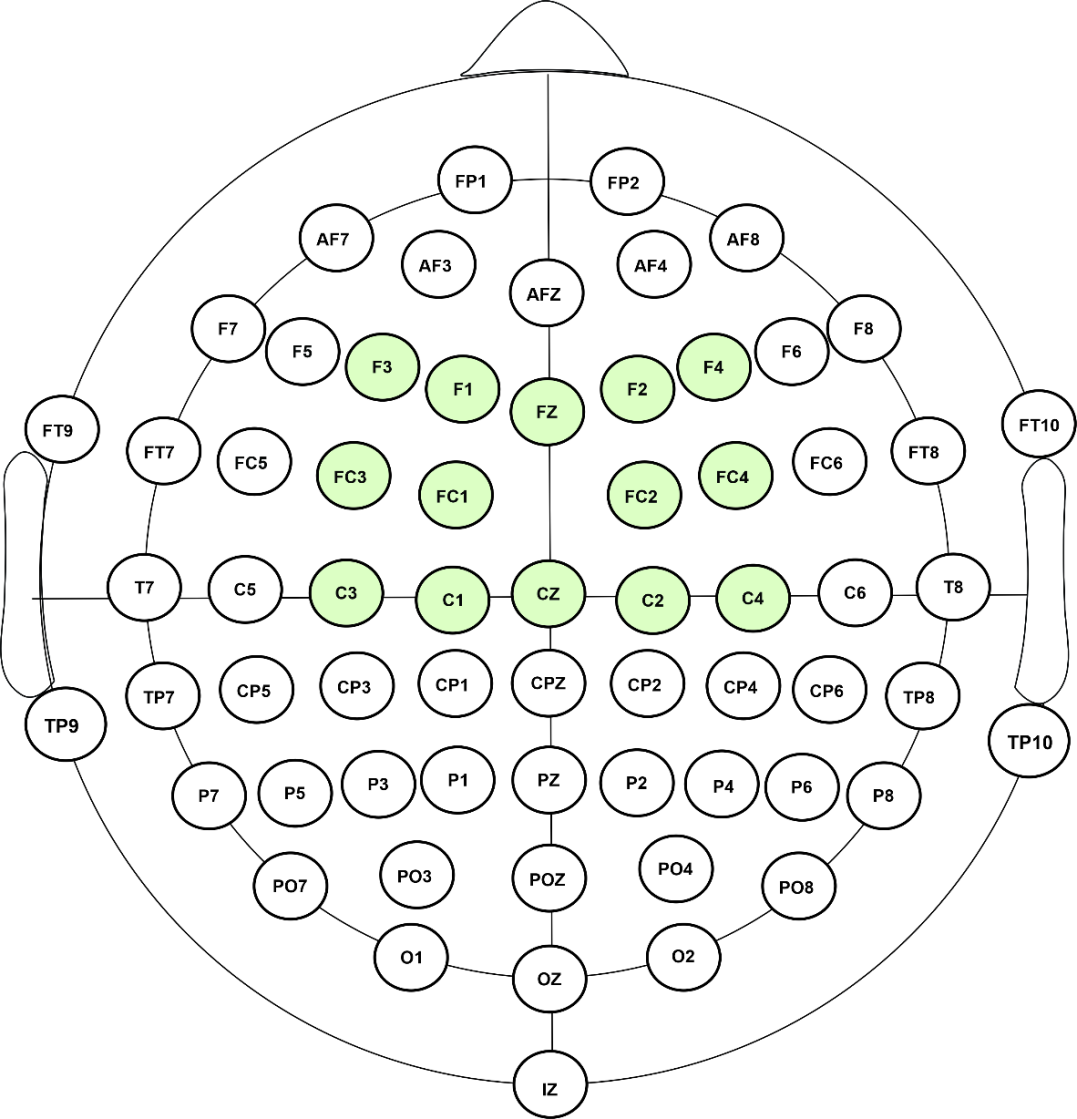
Electrode montage with 64 electrodes used in the experiment. For the analysis, we used the regions of interest (ROI) labelled fronto-central, created by pooling together the electrodes with green filling.

The continuous EEG activity was recorded using a 64-channel recording system (BrainAmp amplifier and BrainVision Recorder software, BrainProducts GmbH) with a sampling rate of 1000 Hz. We mounted 64 electrodes in an electrode cap (actiCAP) with a saline electrolyte solution (see Fig. 3). The electrode FCz was used as a reference.

The data were preprocessed with EEGLAB^52^. Resampling was done at 500 Hz, and high- and low-pass filters were applied (0.1–40 Hz). Channels that were consistently flat or extremely noisy were labelled “bad channels” and removed (M = 2.61). Independent component analysis (ICA) was conducted to correct for artifacts associated with eye movements and heartbeats. After the ICA, eye, heart rate, and muscle (over 98%) components were removed. Afterward, the removed channels were interpolated. The recomposed EEG data were rereferenced to the average activity of all electrodes. The EEG data were subsequently segmented from 1 to 33 s relative to the onset of the stimulus in each condition. The first second is removed to discard evoked responses^26,27^.

After the segmentation of the data, epoch rejection was done, detecting segments that exceeded ± 100 µV. Only participants whose percentage of rejected epochs was below 50% in any of the conditions were included in further data analysis. Accordingly, the data of two participants was excluded. The mean number of retained epochs in the unsyncopated condition was 7.19 out of the possible 8 (SD = 1.15; range: 4–8), and the mean number of retained epochs in the syncopated condition was 7.25 out of the possible 8 (SD = 0.95; range: 4–8). Finally, the remaining epochs were averaged. The preprocessed data sets were exported to Letswave7^53^ for further analysis.

### EEG data analysis

Following the analysis step of the frequency tagging method from Cantiani and colleagues^10^, we first created a region of interest (ROI) by selecting 14 channels from the fronto-central region, creating the ROI called fronto-central (F3, F1, FZ, F2, F4, FC3, FC1, FC2, FC4, C3, C1, CZ, C2, C4; see Fig. 3). The channels were selected based on previous studies on cortical tracking to music and speech rhythms^28^.

**Fig. 4:**
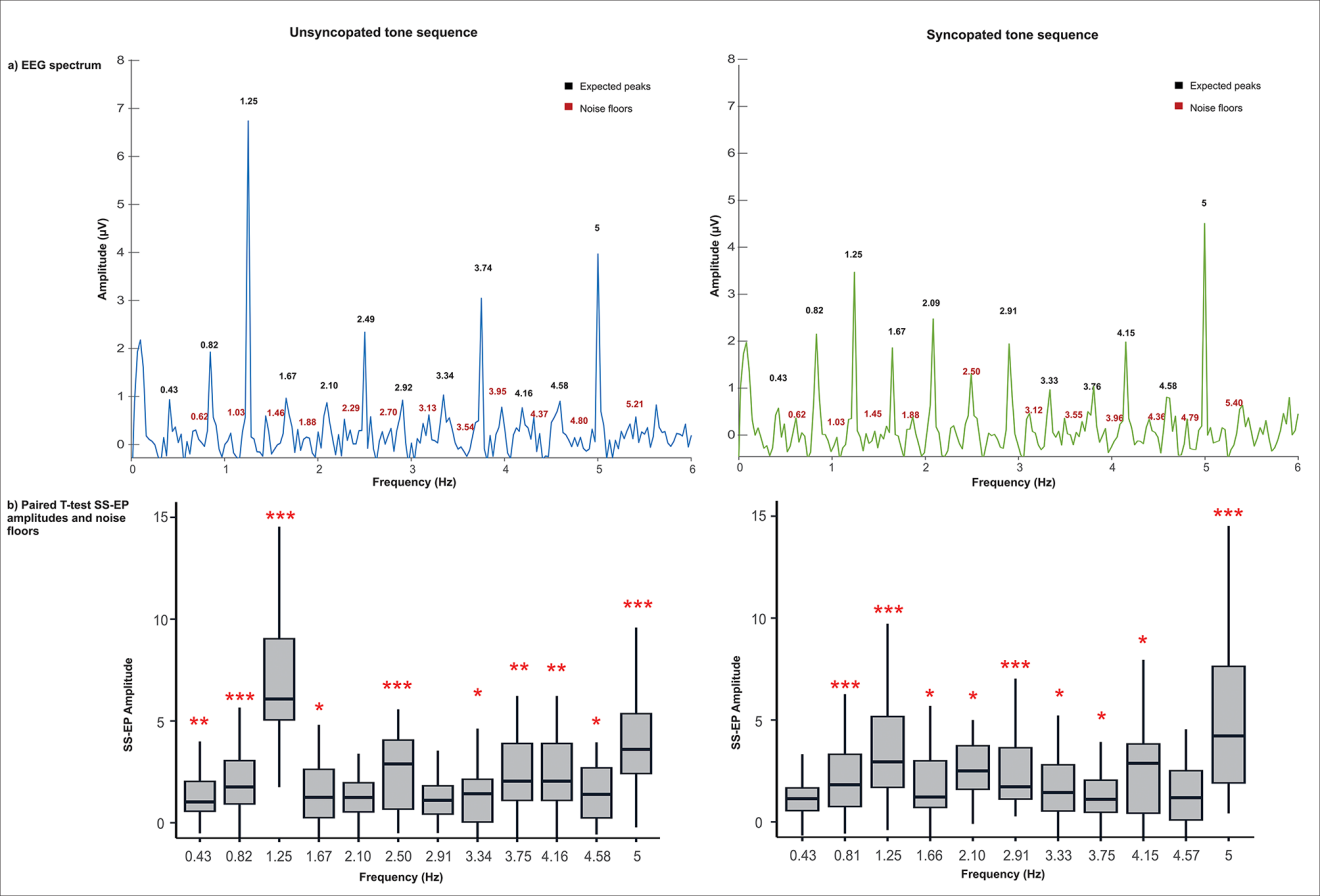
EEG spectrum and paired-sample t test results for unsyncopated and syncopated stimuli. (**A**) Group-level averaged steady-state evoked potentials (SS-EPs) amplitude (µv) expected peaks are labelled in black, and noise floors are labelled in red (*N* = 30). (**B**) Paired sample t test of SS-EP amplitudes (µv) comparing the means of the expected peaks and noise floors for each stimulus. Levels of significant differences are labelled with a red asterisk *, *p < 0.05, **p < 0.01, ***p < 0.001.

Posteriorly, an FFT was applied to compute the frequency spectrum on the ROI. The magnitude of the SS-EPs was calculated in relation to the amplitude of the frequency spectrum at surrounding bins. To remove the residual background noise and determine whether entrainment has occurred, z scores were extracted (i.e., the standard deviation relative to the distribution of the reference interval), considering neighboring bins from − 5––3 and + 3–+5 bins around each frequency bin. Then, for each frequency peak of interest determined by the acoustic sound analysis, the SS-EP magnitudes were extracted within three frequency bins centered around the frequency of interest (for example, for frequency 1.25 Hz, the SS-EP magnitude was extracted considering the frequency range of 1.21–1.28 Hz). To compare enhanced responses to peaks against ‘noise’, we calculated SS‒EP magnitudes for frequencies where we did not expect entrainment (Fig. 1), which were denominated “noise floors” following the temporal envelope of both stimuli^12^. To determine the noise floors for each rhythm, we computed the median between the first two frequency peaks of interest and then computed the relative harmonics. Figure 4 shows the group-level averaged EEG spectrum with the amplitudes for the expected peaks and noise floors (twelve for unsyncopated rhythm and eleven for syncopated rhythm).

### Tapping task data analysis

Two measures were calculated for each trial in both conditions (metronome and music). Synchronization accuracy, synchronization, and continuation tapping consistency. Our focus was on the synchronization phase. We followed the steps described in Kertész and Honbolygó^49^,. The first ten taps were excluded from the analysis. We used Rayleigh’s test to remove trials where tapping did not significantly differ from a random distribution; we marked those trials with NaN for a more accurate average later in the analysis. Outliers in intertap intervals (ITIs) were identified by collecting ITIs for all participants and removing those greater than the third quartile plus three times the interquartile range or smaller than the first quartile minus three times the interquartile range.

Two data analysis methods were used to calculate the difference between each tap and the nearest target beat. To measure tapping accuracy, the mean of the absolute values of differences was divided by the tempo of the music, resulting in a variable that shows the deviation from the reference as a percentage. A value of 0 indicates total synchrony with the stimulus. To calculate tapping consistency, circular statistics were applied^54^. A resultant vector (R) is calculated from all taps in a single trial, where the R vector’s length showed a participant’s tapping consistency, with a hypothetical value of 1 indicating total consistency and 0 indicating total inconsistency. The individual tapping asynchronies were converted into points on a unit circle, with each point representing the distance from the nearest target beat. A value of 0 degrees on the circle indicates a perfectly timed tap.

After calculating consistency and asynchrony degree for each participant trial, we obtained six values corresponding to the combination of two stimuli (metronome, music) and three tempi (80, 120, 150 bpm). We then averaged those values over all trials, separately for two stimuli and three tempi. For later correlational analysis, we created two variables, one for consistency and one for asynchrony averaging across conditions and tempi^49,55^.

### Statistical analysis

#### EEG task

We first conducted a three-way repeated-measures ANOVA with the open-source statistical software JASP (Version 0.18.3, JASP Team, 2024), including as factors, Rhythm (unsyncopated vs. syncopated), Frequency of interest (peak vs. noise) and Spectrum, this factor included five levels: low (0.43 Hz), middle low (1.25 Hz), middle (2.50 Hz for the unsyncopated and 2.10 Hz for the syncopated rhythm), middle high (3.75 Hz), and high (5 Hz). Thus, the complete ANOVA design for the analysis was Rhythm*Frequency of interest*Spectrum. Greenhouse–Geisser correction was applied for factors or interactions where Mauchly’s test indicated that the assumption of sphericity had been violated. Second, we ran paired-sample t tests to compare the SS‒EP magnitudes at the expected peak to those of the noise floors. The false discovery rate (FDR)^56^ was applied to correct for multiple comparisons (FDR < 0.05) in the paired-sample t tests (twelve comparisons for unsyncopated and eleven comparisons for syncopated).

#### Behavioral measures

We used the open-source statistical software JASP (Version 0.19.3, JASP Team, 2025) to run Pearson’s and Spearman’s correlation as required to assess the relationships among the SS-EP magnitudes, tapping task measures (consistency and asynchrony), and two musical background variables (number of instruments and number of years of music education) and counting span average^56^. Moreover, we ran multiple linear regression analysis using the stepwise method to predict tapping consistency and tapping asynchrony, including the averaged unsyncopated SS-EP amplitudes, the averaged syncopated SS-EP amplitudes, number of musical instruments, years of musical education and counting span average as covariates.

## Results

### Steady-state evoked potentials

The group-level averaged SS-EPs for the two rhythms are presented in Fig. 4, including the corrected p values for paired-sample t tests. As expected, the SS-EP amplitudes at 1.25 Hz, corresponding to the beat frequency, were significantly different from those at noise floors, as were those at 2.10/2.50 and 5 Hz. For the SS-EP at 0.43 Hz, there was no significant difference from noise in the syncopated rhythm.

**Fig. 5:**
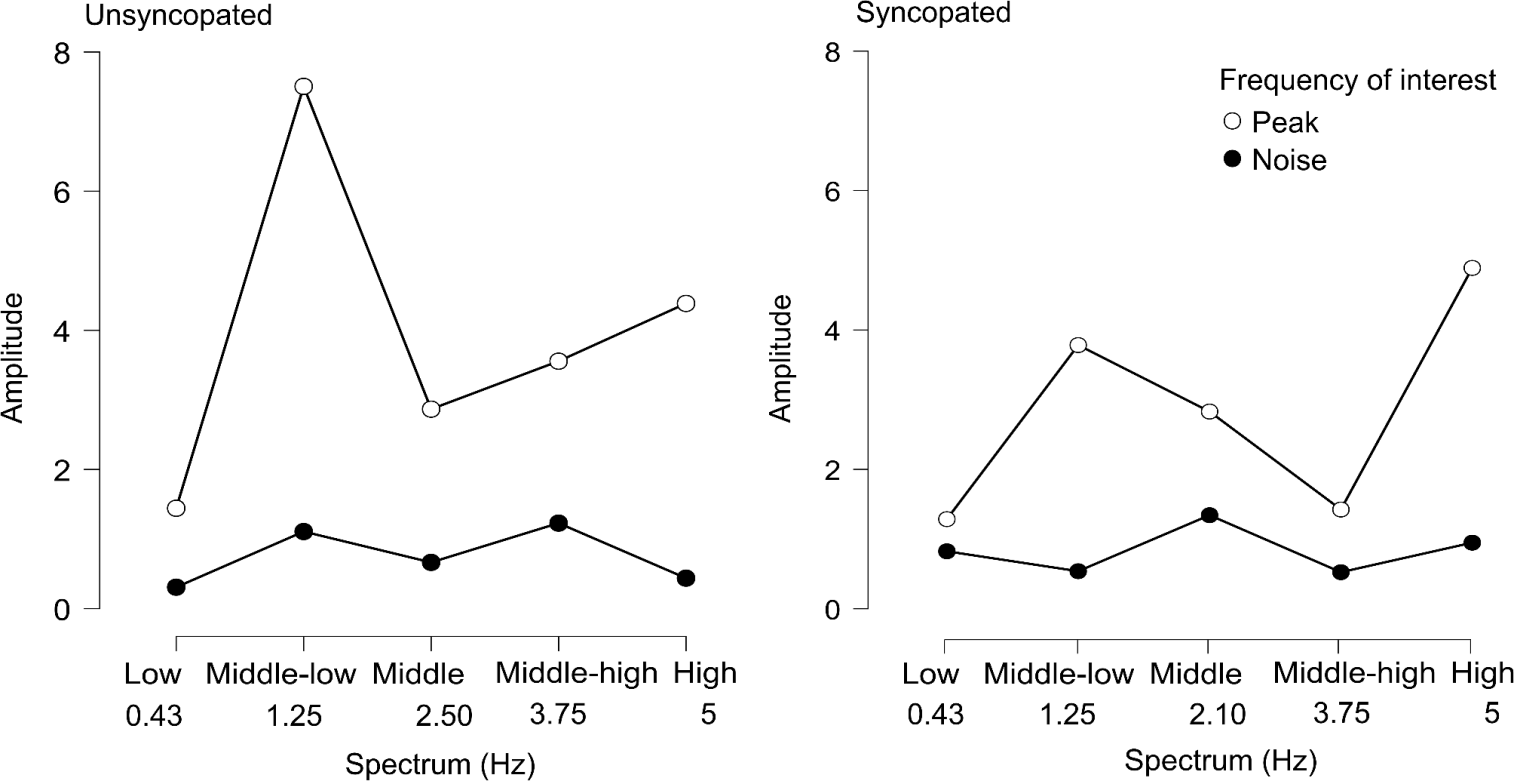
Descriptive plot of repeated-measures ANOVA. The ANOVA design for the analysis was Rhythm*Frequency of interest*Spectrum. The plot shows amplitude differences between unsyncopated and syncopated stimuli through the different spectrum frequencies. Compared with noise floors, beat-related frequencies (1.25, 2.10, 2.50 and 5 Hz) presented larger amplitudes for both stimuli. SS-EP amplitudes and noise floors at 0.43 Hz did not significantly differ between the stimuli. SS-EP amplitudes and noise floors at 3.75 Hz did not significantly differ among the syncopated stimuli.

A repeated-measures ANOVA revealed a main effect of Rhythm, F(1,29) = 9.094, *p* = 0.005, n^2^ = 0.008, Frequency of interest F(1,29) = 148.618, *p* < 0.001, n^2^ = 0.199, and Spectrum factor, F(3.02, 87.48) = 13.232, *p* < 0.001, n^2^ = 0.073. The analysis also revealed a significant interaction between Rhythm and Frequency of interest, F(1,29) = 7.515, *p* = 0.010, n^2^ = 0.010; a significant interaction between Rhythm and Spectrum, F(3.10,89.941) = 10.110, *p* < 0.001, n^2^ = 0.033; and a significant interaction between Frequency of interest and Spectrum, F(3.12,90.32) = 12.825, *p* < 0.001, n^2^ = 0.068. Finally, we found a significant three-way interaction between Rhythm*Frequency of interest*Spectrum, F(4, 116) = 2.767, *p* = 0.031, n^2^ = 0.009. Post hoc Bonferroni analysis revealed larger amplitudes for the unsyncopated rhythm than for the syncopated rhythm across the different chosen spectrum frequencies. As can be seen in Fig. 5, we also found significant differences between noise and peak amplitudes, with larger amplitudes for the latter being found for the beat frequency (1.25 Hz) and related harmonics at 2.50, 3.75, and 5 Hz in the unsyncopated rhythm and for the syncopated rhythm at the beat frequency (1.25 Hz) and related harmonics at 2.10 and 5 Hz. No significant differences were found at 0.43 Hz harmonics for either rhythm. Finally, the amplitudes for the expected peaks varied across the different chosen spectrum frequencies, in which the largest amplitudes corresponded to 1.25 Hz and 5 Hz.

### Relationships between SS-EPs and behavioral measures

To identify differences in interactions with behavioral measures based on rhythm, we first calculated the average SS-EP amplitudes at the expected beat frequency and other expected peaks, including those frequencies for which paired t test revealed significant differences from noise (see Fig. 4), for the unsyncopated and syncopated rhythms separately. To identify overall interactions, we calculated a third variable with the average SS-EP amplitude for both syncopated and unsyncopated rhythms.

We first conducted correlation analysis with the tapping task measures of consistency and asynchrony to identify interactions between SS-EPs amplitudes, sensorimotor synchronization skills and musical background variables. The correlation matrix can be seen in Table 1. This correlation analysis helped us to identify the most suitable SS-EPs amplitude variables and musical background variables to avoid multicollinearity when conducting the multiple linear regression analysis.

**Table 1:** Correlation matrix of SSEP variables and behavioural variables. Mean (M) and standard deviations (SD) of each variable are included. *N* = 30.

Variable	M	SD	1	2	3	4	5	6	7	8
1. Unsyncopated averaged SSEP	3.02	1.13	–							
2. Syncopated averaged SSEP	2.69	0.99	0.316	–						
3. Unsyncopated + Syncopated averaged SSEP	2.85	0.86	0.836***	0.784***	–					
4. Averaged tapping consistency	0.64	0.16	-0.449*	-0.285	-0.410*	–				
5. Averaged tapping asynchrony	0.09	0.02	0.405*	0.308	0.376*	− 0.829***	–			
6. Number of musical instruments ^a^	1.17	1.2	− 0.406*	-0.333	− 0.462*	0.327	− 0.357	–		
7. Years of musical education	4.40	4.91	− 0.245	-0.270	− 0.316	0.070	− 0.094	0.792***	–	
8. Counting span	4.39	0.80	− 0.094	0.020	− 0.050	0.378*	− 0.206	0.217	0.227	–

*p < 0.05, ***p < 0.001.

^a^Spearman’s correlation was used for this ordinal variable.

#### Predicting tapping performance from SS-EP amplitudes

To predict tapping performance (consistency and asynchrony), the unsyncopated and syncopated averaged SS-EP amplitude, counting span average, years of musical education and total number of musical instruments play(ed) as covariates were entered in a series of multiple linear regression analysis using stepwise method.

The first model was aimed to predict tapping consistency. The results showed that tapping consistency was best predicted by the average unsyncopated SS-EP amplitude and counting span average F(2,27) = 6.235, *p* = 0.006, R^2^ = 0.316, R^2^_Adjusted_ = 0.265. The R^2^ value illustrates that the model can account for 27% of the tapping consistency variability. An analysis of standard residuals revealed that the data contained no outliers (Std. Residual Min = -2.52, Std. Residual Max = 2.31). The histogram of standardised residuals showed a normal distribution of errors. The scatterplot of standardised predicted values showed a random distribution therefore the assumptions of homogeneity of variance and linearity were met. The assumption of collinearity indicated that multicollinearity was not a concern (Averaged unsyncopated SS-EPs, Tolerance = 0.99, VIF = 1.009; Counting average span, Tolerance = 0.99, VIF = 1.009). The tapping consistency can be predicted by the following regression equation: Tapping consistency = 0.52 + (− 0.060 * unsyncopated average SSEP) + (0.068 * counting average span).

The second model was aimed to predict tapping asynchrony. The results showed that tapping asynchrony was best predicted by the average unsyncopated SS-EP amplitude F(1,28) = 5.486, *p* = 0.027, R^2^ = 0.164, R^2^_Adjusted_ = 0.134. The R^2^ value illustrates that the model can account for 13% of the tapping asynchrony variability. An analysis of standard residuals revealed that the data contained no outliers (Std. Residual Min = -2.510, Std. Residual Max = 1.582). The histogram of standardised residuals showed a normal indicated that the distribution of errors was approximately normal, as did the P–P plot of standardised residuals. The scatterplot of standardised predicted values showed a random distribution therefore the assumptions of homogeneity of variance and linearity were met. The assumption of collinearity indicated that multicollinearity was not a concern (Averaged unsyncopated SS-EPs, Tolerance = 1.000, VIF = 1.000).

The tapping asynchrony can be predicted by the following regression equation: Tapping asynchrony = 0.058 + (0.009 * unsyncopated average SSEP).

Table 2 shows the regression coefficients for the final models predicting tapping performance.

**Table 2:** Regression coefficients for the final models predicting tapping performance, consistency and asynchrony.

Variable	Tapping consistency				Tapping asynchrony			
	B	SE	β	*p*	B	SE	β	*p*
Constant	0.516	0.165	–	0.004	0.058	0.013		< 0.001
Unsyncopated averaged SSEP	− 0.060	0.023	− 0.418	0.015	0.009	0.004	0.405	0.027
Counting span	0.068	0.032	0.339	0.043	–	–	–	–
R^2^ _Adjusted_	0.27**			R^2^	0.13*			

*p < .05, **p < .01.

## Discussion

The present study aimed to replicate the findings on neural entrainment to unsyncopated and syncopated rhythms. Moreover, it also aimed to identify whether neural entrainment patterns in addition to working memory and musical background could explain finger-tapping performance variability. In our study, EEG data was recorded while participants listened to unsyncopated and syncopated tone sequences, and to ensure attention, they were asked to detect tempo changes at the end of each trial. The experimental stimuli and procedure were a replication of the paradigm of Nozaradan and colleagues^26^. The participants also completed a finger-tapping task with six trials including metronome and music stimuli, a counting span task, and a musical background questionnaire.

### Neural entrainment to the beat

Our first aim was to replicate previous results on neural entrainment using the frequency tagging method, we found that participants in the fronto-central sites were cortically tracking the rhythmic contour of unsyncopated and syncopated tone sequences. The participants showed a greater amplitude at the expected frequency peaks (SS-EPs) based on the stimuli envelope, and these amplitudes were significantly different from the amplitudes from those frequencies labelled noise floors in both rhythms. Based on previous literature, a larger amplitude reflects a more faithful cortical tracking response, frequency-locked to the acoustic envelope of the tone sequences^23,26,27^.

Enhanced sensory responses are associated with attentional fluctuations guided by expectations^30,31^; neural entrainment models suggest that low-frequency neural oscillations are entrained to the rhythmic regularities of external events and that brain oscillations are phase-locked to external rhythms and reset to match high excitability phases to the onset of expected events^33,57^. This allows beat-based predictions to be built; therefore, enhanced sensory responses are expected to reflect heightened synchronization of neural firing patterns that guide attentional resources and facilitate perception^17,28,29^. In the case of music, an enhanced neural response is expected at beat frequency and meter-related frequencies^23,25^.

Our results revealed an enhanced neural response elicited at 1.25 Hz and 5 Hz compared with other expected peaks for both rhythms; this enhanced response is especially interesting in the syncopated rhythm where there is less acoustic energy coming from the stimuli envelope, suggesting that the cortical tracking of the rhythmic contour of tone sequences not only tracks the acoustic energy from stimuli but also reflects internal beat generation. This is in line with neural entrainment models that suggest that neural oscillations in the low-frequency band have preferred rhythms around the beat tempo range^5,14,21^, which allows neural responses to track and enhance at beat frequencies and related meter frequencies; in this sense, SS-EP amplitudes do not merely reflect evoked potentials but rather spontaneous selective neural entrainment to beat and meter. Therefore, our participants were neurally entrained to unsyncopated and syncopated rhythms and generated an internal representation of the beat, corroborating previous findings^23^.

### Individual differences in sensorimotor synchronization skills

Our second aim was to identify whether neural entrainment alongside cognitive resources, specifically working memory and musical background could predict sensorimotor synchronization skills in adults. Tapping consistency was best predicted by the averaged unsyncopated SS-EPs amplitudes and counting span average, while for tapping asynchrony, the averaged unsyncopated SS-EPs amplitudes was the sole predictor.

Interestingly, in our first regression model aimed to predict tapping consistency (i.e., the regularity of the participants’ tapping to the rhythm), the unsyncopated SS-EPs amplitudes had a negative coefficient, which shows that reduced SS-EPs amplitudes to unsyncopated rhythms are associated with higher tapping consistency, while in our second regression model aimed to predict tapping asynchrony (i.e., how inaccurately participants were tapping to the rhythm) the unsyncopated SS-EPs amplitudes had a positive coefficient, which shows that larger SS-EPs amplitudes to unsyncopated rhythms are associated with higher tapping asynchrony.

This indicates that participants with larger SS-EP amplitudes to unsyncopated rhythms, performed worse on the finger-tapping task, exhibiting lower tapping consistency and greater asynchrony. These findings do not align with our hypothesis and differ from those of previous studies, which have shown that participants with stronger sustained entrainment are more accurate and consistent in tapping tasks^27^. It has been suggested that the entrainment of neural oscillations allows participants to build predictions of important events to come; in a tapping task, participants who become entrained to the beat tempo can more easily detect the incoming beat and maintain their tapping tempo, reflecting better tapping synchronization and consistency^21^.

According to our results, working memory was identified as a significant predictor contributing to tapping consistency but not tapping asynchrony. Cognitive resources and rhythm processing skills have been previously linked: specifically working memory has been suggested to contribute to rhythm processing skills by maintaining representations of temporal intervals^44,58^. These temporal representations seem to aid participants in predicting time intervals to maintain tapping consistency, similar to our results, Colley and colleagues^59^ found that higher working memory capacity was associated with better anticipation of beat onsets (beat tracking) but working memory capacity was not associated to asynchrony. The authors suggested that working memory has a role in predicting time intervals while attentional systems might play a more important role in detecting temporal discrepancies between the tapping and the beat.

Our study suggests that participants with greater counting span capacity are better at maintaining internal temporal representations and adjusting them to an external rhythm, resulting in more consistent tapping. This is supported by previous research showing that working memory predicts lower variability in rhythm production abilities^60^ and that accuracy of temporal control of movements depends on working memory load demands^61^.

In addition, musical background was also expected to contribute to our regression models. However, the number of musical instruments played, and years of musical education, the two variables that we used to characterize musical background, were not significant predictors in our models. Musical background has been consistently associated with better synchronization skills in previous research^38,39^. However, previous studies have also shown similar performance in phase correction between amateur musicians and non-musicians during a finger-tapping task^62^, as well better tapping consistency and reduced asynchrony has been found in percussionists compared to other musicians^39,63^ which can suggest that the type of musical instrument played can impact tapping performance. In our study the majority of participants were not professional musicians, and neither were active musicians, our population reflect general sensorimotor synchronization skills in adulthood, where musical background as measured by the previously described variables seems not to be a predictor of tapping performance.

The unexpectedly negative relationship between SS-EP amplitudes and tapping consistency and synchronization, must be further discuss, we can interpret our results following the previously introduced types of neural prediction mechanisms, beat-based and memory-based expectations^28^.

As described in the Introduction, beat-based expectations arise from a periodic input, detecting a regular beat and enhancing phase-locked neural response evidenced in the neural entrainment to the beat, and this can be seen in our results of enhanced SS-EPs amplitudes to unsyncopated and syncopated rhythms at beat frequencies and related meters. In contrast, memory-based expectations rely on learned associations between cues, temporal intervals, and events, requiring attentional and memory resources during pattern formation, and reducing neural responses once the patterns have been established^28^.

### Beat-based and memory-based predictions in neural entrainment and sensorimotor synchronization

In the context of our results, the stimuli used for the SS-EPs task were tone sequences that induce beat perception; they were highly periodic and drove automatic neural entrainment processes led by beat-based expectations, it falls in line that it was the unsyncopated SS-EP amplitude that resulted in a significant predictor, this rhythm was considered as “explicit” since the beat coincided with the tone, therefore facilitating neural entrainment, evidenced on the larger SS-EP amplitudes for this rhythm. In contrast, the tapping task requires not only beat perception but also rhythm production. This same beat-based expectation might not have been the best strategy to maintain tapping consistency and asynchrony, as it was a goal-directed behavior that required cognitive control. Consequently, participants who were better at neurally entraining to rhythmic contours might not necessarily have better performance in this particular tapping task.

On the other hand, memory-based predictions, because they require higher order cognitive processes such as working memory, may be better suited to meet the demands of rhythm production tasks. Working memory creates and maintains representations of temporal and motor patterns that can be used later for error correction and timing adjustment during tapping performance^59^. Working memory has also been found to predict rhythm production abilities in patients with neurocognitive disorders, but working memory was not a predictor of beat perception abilities, showing a dissociation between task demands on cognitive resources^60^.

Similar unexpected results in rhythm processing were also reported in a study by McPherson and colleagues^64^, where spontaneous tapping without stimulus presentation was measured via phase locking values (PLVs), reflecting how constant participants’ tapping was with their own tempo. The participants were classified into two groups: those with a higher PLV and less variability in their tapping were labelled high rhythmicity, and those with a low PLV and more variability in their tapping were labelled low rhythmicity. It was predicted that those with high rhythmicity would perform better in a synchronization‒continuation tapping paradigm since high- rhythmicity participants are suggested to have greater sustained entrainment to rhythmic stimuli. However, they reported the opposite trend: the high-intensity rhythmicity group tapped with less accuracy than the low-intensity rhythmicity group did, which showed greater accuracy; high-intensity rhythmicity participants also showed early entrainment decay, indicating that they deviated from the prompt beat sooner than did the low-intensity rhythmicity participants. High rhythmicity participants’ performance was biased by their generated pulses, and they relied less on external pulses.

Based on our results, we hypothesize that beat-based predictions lead participants’ higher entrainment to tone sequences; however, this type of prediction may not be the most effective for enhancing consistency and synchrony. Those with smaller SS-EP amplitudes and better working memory capacity are expected to be more consistent with their tapping, and they might rely more on memory-based predictions. This means that the stimuli characteristics do not necessarily entrain them, but they have been able to build temporal patterns that can help them make predictions of important events to come, which has been associated with attenuation of neural responses, as has been observed in the alpha band^35^.

Further support can be found on previous studies on these two types of prediction mechanisms. Bouwer and colleagues^28^ aimed to differentiate between these two types of expectations found that while both beat-based and memory-based expectations facilitated target detection and elicited similar auditory responses to patterns, only beat-based expectations showed a reduced target detection and enhanced auditory responses for unexpected events. Moreover the presence of beat-based expectations did not facilitate memory-based expectations to periodic sequences, on the contrary these two types of predictions seemed to interfere with each other, leading to reduced auditory responses and target detection, which suggest that even though the two types of predictive mechanisms have different computations they might share a limited capacity for temporal processing.

Furthermore, Bouwer and colleagues^36^, found persistent neural entrainment to beat-based stimuli even during silent intervals, while for the pattern-based stimuli there was neither enhanced power at the inherent frequency, nor sustained entrainment during silent intervals, suggesting that neural entrainment does not seem to support pattern-based expectations; instead, pattern-based expectations showed a contingent negative variation (CNV) negative ERP component before expected events.

Based on this, we suggest that participants with a stronger neural entrainment could not be guided by memory-based predictions, as there seems to be a shared limited capacity for temporal processing, and as a result they had enhanced neural responses during the SS-EP task but the neural entrainment may have reduced the flexibility needed in the rhythm production task. On the other hand, participants with reduced neural entrainment might have built predictions by learning temporal patterns, relying on memory-based predictions, and therefore showing reduced neural responses, mechanism that gave them an advantage in the finger tapping task. Nonetheless, we cannot provide evidence that participants used only one type of expectation, the stimuli used for the SS‒EP task, as well as the stimuli used for the tapping task could also have elicited both beat-based and memory-based predictions, and these types of expectations are more likely to interact and modulate each other to respond to environmental demands^34^.

### Limitations and future directions

One of the limitations of our study is that we did not include a behavioral measure of beat perception. The stimuli used for the EEG task were rhythmic sequences highly periodic and designed to elicit entrainment, whereas for the tapping task, we used metronome sounds and instrumental versions of popular songs, and some participants might have been more familiar with them. As the nature of the stimuli used for the EEG task and tapping task differ, the neural responses could also differ; in further research, more complex musical stimuli could be used in the EEG task, introducing tempo changes, as they would require intentional synchronization from the participant, allowing a more appropriate comparison between performances. Additionally, a standardized questionnaire to measure musical background as well as group comparisons between musicians and non-musicians could provide clearer information about the contributions of musical background in tapping performance.

Another limitation is the correlational nature of our study, the relationship between SS-EP, working memory, and tapping performance was established in a multiple regression analysis, which does not allow to draw causal conclusions. Nonetheless, the unexpected findings on the association between these variables might drive future studies which could explore casual links.

Additional research is needed with paradigms including stimuli that allow us to differentiate between beat-based and memory-based prediction and how these interact on rhythm processing on the neural and behavioral level to further unveil individual differences in rhythm processing skills.

Lastly, understanding individual differences in rhythm processing and its multidimensionality has important implications for the adequate identification of rhythm processing impairments^65,66^. Atypical rhythm processing/production has been suggested to be a potential risk factor for developmental disorders and speech and language disorders^67^, such as dyslexia^68,69^ stuttering^54^ and ADHD^70^. Therefore, the cognitive and neural mechanisms of rhythm processing should remain an active research area.

## Conclusion

This study replicated previous findings on neural entrainment to rhythmic patterns and explored individual differences in neural entrainment, working memory, and musical background as predictors of sensorimotor synchronization skills. The findings revealed that while enhanced steady-state evoked potentials (SS-EPs) indicated faithful cortical tracking of both unsyncopated and syncopated rhythms at beat and meter frequencies, they were unexpectedly associated with poorer tapping performance, less consistency, and greater asynchrony among participants. This unexpected direction challenges prior assumptions that stronger neural entrainment enhances sensorimotor synchronization accuracy and consistency. In our results participants with smaller SS-EP amplitudes and greater working memory capacity demonstrated better tapping consistency. Our findings indicate that SS-EP amplitudes reflect neural entrainment patterns elicited by beat-based predictions during a passive listening task but suggest that memory-based predictions and cognitive resources such as working memory and cognitive control might be required during a rhythm production task. Furthermore, the unexpected pattern found can also support the multidimensionality of rhythm processing skills, for instance, participants who perform better at beat detection tasks do not necessarily perform better at rhythm production tasks. The difference between the neural and behavioral measures could be attributed to differences in the skills needed to solve them. The results revealed the complex relationship between neural entrainment and sensorimotor synchronization and highlighted the complexity of rhythm processing, where distinct neural mechanisms—beat-based and memory-based—may support the multidimensionality of rhythmic abilities influenced by individual differences such as working memory.

## Electronic supplementary material

Below is the link to the electronic supplementary material.

Supplementary Material 1

## Data Availability

The data that support the findings of this study are available from the following repository: https://osf.io/4b58c/?view_only=874cd91c91e34a06b8467595fc5d518c.
