# Supplementary material for: Neural entrainment to the beat and working memory predict sensorimotor synchronization skills
Source: Sci Rep. 2025 Mar 26;15:10466. doi: 10.1038/s41598-025-93948-9 (PMC11947151; doi:10.1038/s41598-025-93948-9)
Supplement: Supplementary file 1 — Supplementary Material 1 [file 41598_2025_93948_MOESM1_ESM.docx]

Supplementary materials

Neural entrainment to the beat and working memory predict sensorimotor synchronization skills

María de Lourdes Noboa*^1,2,3^ Csaba Kertész ^2^, Ferenc Honbolygó ^2,3^

^1^ Doctoral School of Psychology, ELTE Eötvös Loránd University, Budapest, Hungary

^2^ Institute of Psychology, ELTE Eötvös Loránd University, Budapest, Hungary

^3^ Brain Imaging Centre, Research Centre for Natural Sciences, Budapest, Hungary

Corresponding author:

María de Lourdes Noboa

Institute of Psychology of the Faculty of Education and Psychology

Eötvös Loránd University

Izabella u. 46, 1064, Budapest – Hungary

Email: [noboa.maria@ppk.elte.hu](mailto:noboa.maria@ppk.elte.hu)

Phone: +36 304324394

*Three-way Repeated Measures Analysis of Variance for the SS-EPs amplitudes*

| **Effects** | **Sphericity Correction** | **F** | **df** | **p** | **η²** |
| --- | --- | --- | --- | --- | --- |
| Rhythm | None | 9.094 | 1.000 | 0.005 | 0.008 |
| Residuals | None |  | 29.000 |  |  |
| Frequency of interest | None | 148.618 | 1.000 | < .001 | 0.199 |
| Residuals | None |  | 29.000 |  |  |
| Spectrum | None | 13.232 ^a^ | 4.000 ^a^ | < .001 ^a^ | 0.073 |
|  | Greenhouse-Geisser | 13.232 | 3.017 | < .001 | 0.073 |
| Residuals | None |  | 116.00 |  |  |
|  | Greenhouse-Geisser |  | 87.480 |  |  |
| Rhythm ✻ Frequency of interest | None | 7.515 | 1.000 | 0.010 | 0.010 |
| Residuals | None |  | 29.000 |  |  |
| Rhythm ✻ Spectrum | None | 10.110 ^a^ | 4.000 ^a^ | < .001 ^a^ | 0.033 |
|  | Greenhouse-Geisser | 10.110 | 3.101 | < .001 | 0.033 |
| Residuals | None |  | 116.00 |  |  |
|  | Greenhouse-Geisser |  | 89.935 |  |  |
| Frequency of interest ✻ Spectrum | None | 12.825 ^a^ | 4.000 ^a^ | < .001 ^a^ | 0.068 |
|  | Greenhouse-Geisser | 12.825 | 3.115 | < .001 | 0.068 |
| Residuals | None |  | 116.00 |  |  |
|  | Greenhouse-Geisser |  | 90.324 |  |  |
| Rhythm ✻ Frequency of interest ✻ Spectrum | None | 2.767 | 4.000 | 0.031 | 0.009 |
| Residuals | None |  | 116.00 |  |  |
| *Note.* ᵃ Mauchly's test of sphericity indicates that the assumption of sphericity is violated (p < .05). | | | | | |
